# The Balancing of Peroxynitrite Detoxification between Ferric Heme-Proteins and CO_2_: The Case of Zebrafish Nitrobindin

**DOI:** 10.3390/antiox11101932

**Published:** 2022-09-28

**Authors:** Giovanna De Simone, Andrea Coletta, Alessandra di Masi, Massimo Coletta, Paolo Ascenzi

**Affiliations:** 1 Dipartimento di Scienze, Università Roma Tre, 00146 Roma, Italy; 2 IRCCS Fondazione Bietti, 00198 Roma, Italy

**Keywords:** *Danio rerio* heme-protein, effect of CO_2_, kinetics, peroxynitrite detoxification, tyrosine protection, zebrafish nitrobindin

## Abstract

Nitrobindins (Nbs) are all-β-barrel heme proteins and are present in prokaryotes and eukaryotes. Although their function(s) is still obscure, Nbs trap NO and inactivate peroxynitrite. Here, the kinetics of peroxynitrite scavenging by ferric *Danio rerio* Nb (*Dr*-Nb(III)) in the absence and presence of CO_2_ is reported. The *Dr*-Nb(III)-catalyzed scavenging of peroxynitrite is facilitated by a low pH, indicating that the heme protein interacts preferentially with peroxynitrous acid, leading to the formation of nitrate (~91%) and nitrite (~9%). The physiological levels of CO_2_ dramatically facilitate the spontaneous decay of peroxynitrite, overwhelming the scavenging activity of *Dr*-Nb(III). The effect of *Dr*-Nb(III) on the peroxynitrite-induced nitration of L-tyrosine was also investigated. *Dr*-Nb(III) inhibits the peroxynitrite-mediated nitration of free L-tyrosine, while, in the presence of CO_2_, *Dr*-Nb(III) does not impair nitro-L-tyrosine formation. The comparative analysis of the present results with data reported in the literature indicates that, to act as efficient peroxynitrite scavengers in vivo, i.e., in the presence of physiological levels of CO_2_, the ferric heme protein concentration must be higher than 10^−4^ M. Thus, only the circulating ferric hemoglobin levels appear to be high enough to efficiently compete with CO_2_/HCO_3_^−^ in peroxynitrite inactivation. The present results are of the utmost importance for tissues, like the eye retina in fish, where blood circulation is critical for adaptation to diving conditions.

## 1. Introduction

Nitrosative stress plays a key role in the etiology of human diseases, such as atherosclerosis, inflammation, cancer, and neurological diseases, being particularly relevant in the onset of retinopathies and glaucoma [1,2,3]. In fact, reactive nitrogen species (RNS) can cause protein, DNA, and lipid nitration, impairing their functions [4,5,6,7,8,9,10,11]. One of the most potent biological nitrosative agents is peroxynitrite (ONOO^−^), which is produced when nitric oxide (^●^NO) and superoxide (^●^O_2_^−^) are combined at extremely rapid rates [4,5,9,10,11,12,13]. ONOO^−^ diffuses through membrane anion channels as well as the conjugated peroxynitrous acid ONOOH (p*K*_a_ = 6.9) [13]. ONOO^−^ is relatively stable, while ONOOH decays rapidly, with an apparent half-life of 1-2 s at physiological pH, yielding ~70% nitrate (NO_3_^−^) and H^+^, and ~30% nitrogen dioxide (^●^NO_2_) and hydroxyl (^●^OH) radicals via the homolysis of the O–O bond. The secondary reactions of ^●^NO_2_ and O^●−^ lead to NO_2_^−^ and O_2_ [4,9,10,11,12,13]. ONOO^−^ reacts with biomolecules mainly by a direct reaction or immediately after ONOOH is homolyzed to ^●^NO_2_ and ^●^OH [4,9,10,11,12,13].

**Scheme 1:**
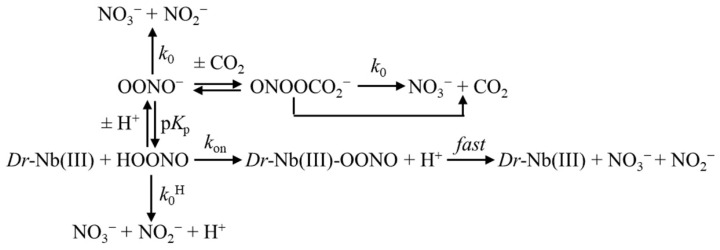
*Dr*-Nb(III)-mediated isomerization of peroxynitrite in the absence and presence of CO_2_ (representing the CO_2_/HCO_3_^−^/CO_3_^2−^ system). The disappearance of the pH-dependent ONOO^−^/ONOOH species is characterized by the absorption decrease at 302 nm. For the sake of clarity, only the final products of the peroxynitrite inactivation mechanisms (indicated by *k*_0_ and *k*_0_^H^) are reported, while the intermediate species are described in the text.

At the end of the last century, carbon dioxide (CO_2_) was reported to react with ONOO^−^ [14,15]. Since the CO_2_ concentration is relatively high in vivo (~1.2 × 10^−3^ M at physiological pH in equilibrium with ~2.4 × 10^−2^ M HCO_3_^−^ with a p*K*_a_ ≈ 6.3 at 25.0 °C), most of the ONOO^−^ rapidly reacts with the CO_2_/HCO_3_^−^ species (depending on pH), leading mostly to the formation of 1-carboxylato-2-nitrosodioxidane adduct (ONOOCO_2_^−^), which displays an apparent half-life of 0.5–3 ms. This compound further decays (via homolysis of the O–O bond), yielding the reactive species trioxocarbonate^●−^ (CO_3_^●−^) and ^●^NO_2_ (3% to 35%), which then proceed towards CO_2_ and NO_3_^−^ (or by direct yielding of NO_3_^−^ and CO_2_ (65% to 90%) [9,11,12,13,14,15,16,17,18,19,20,21]). As a whole, the complexity of the peroxynitrite inactivation mechanism can be sketched, as is shown in Scheme 1 [13,22,23,24,25,26,27,28,29,30,31,32,33,34,35,36,37,38,39,40,41], where *Dr*-Nb(III) represents the contribution of heme proteins.

Moreover, the reaction of ONOO^−^ with CO_2_/HCO_3_^−^/CO_3_^2−^ redirects the ONOO^−^/ONOOH-based oxidation of aromatic and aliphatic amino acid residues, facilitating the nitration of tyrosine and tryptophan and limiting the oxidation of methionine and cysteine [5,9,10,11,12,13,14,15,20,21,42,43,44,45]. Remarkably, ^●^NO_2_ and CO_3_^●−^ radicals are much stronger oxidant species than their precursors ^●^NO, O_2_^●−^, and ONOO^−^/ONOOH [9,10,11,12,13,20,21,46].

Tyrosine nitration through the peroxynitrite pathway (see Scheme 1) is operative in most fish, including zebrafish (*Danio rerio*), under stressed conditions [47,48]. Further, the effect of the enrichment of CO_2_ in the atmosphere brings about the raising of CO_2_ levels also in the oceans [49]. Although the increased levels of CO_2_ create only limited behavioral effects, they may significantly affect the body response to external insults. In addition, the increased concentration of CO_2_ in the blood is of particular relevance in fish, for which retinal circulation is of crucial importance due to the high hydrostatic pressure of the environment where they live [50,51].

Here, the kinetics of ONOO^−^/ONOOH (hereafter peroxynitrite) inactivation by all-β-barrel ferric *Danio rerio* nitrobindin (*Dr*-Nb(III)), in the absence and presence of CO_2_, are reported and analyzed in parallel with those of all-α-helical globins. The present results open a question on the competition between the protecting activity of heme proteins (which inactivate the damaging reactions of peroxynitrite on L-tyrosine nitration) and the non-protecting action of CO_2_/HCO_3_^−^, which indeed depends on the levels of ferric heme-proteins. Thus, the levels (and the scavenging activity) of most heme proteins might be too low to act as peroxynitrite scavengers in vivo; only circulating ferric hemoglobin (Hb(III)) levels appear to be high enough to efficiently compete with CO_2_/HCO_3_^−^/CO_3_^2-^ in peroxynitrite inactivation.

## 2. Materials and Methods

### 2.1. Materials

*Dr*-Nb(III) was cloned, expressed, and purified as already reported [52]. The *Dr*-Nb(III) concentration was determined spectrophotometrically by measuring the absorbance at 407 nm (ε = 1.57 × 10^5^ M^−1^ cm^−1^) [52]. Apo-*Dr*-Nb was prepared, as already reported [53]. Peroxynitrite was obtained from Cayman Chemical (Ann Arbor, MI, USA). The concentration of peroxynitrite was determined spectrophotometrically at 302 nm (ε = 1.705 × 10^3^ M^−1^ cm^−1^) [12]. L-tyrosine (obtained from Merck KGaA, Darmstadt, Germany) was dissolved in 5.0 × 10^−2^ M Bis-Tris propane buffer, at pH 7.0 and 22.0 °C; the final L-tyrosine concentration was 1.0 × 10^−4^ M [25,30,36,39]. All the other chemicals were purchased from Merck KGaA (Darmstadt, Germany). All chemicals were of analytical grade and were used without further purification.

### 2.2. Methods

In the absence and presence of CO_2_, peroxynitrite isomerization by *Dr*-Nb(III) and apo-*Dr*-Nb was investigated at pH 5.8, 7.0, and 8.5 (5.0 × 10^−2^ M Bis-Tris propane buffer) and 22.0 °C, under anaerobic conditions. In the presence of CO_2_, peroxynitrite isomerization by *Dr*-Nb(III) and apo-*Dr*-Nb was investigated by adding NaHCO_3_ (final concentration, 5.0 × 10^−1^ M) to the *Dr*-Nb(III) and apo-*Dr*-Nb solutions; this NaHCO_3_ concentration corresponds to different concentrations of CO_2_, HCO_3_^−^, and CO_3_^2-^ depending on pH. After the addition of NaHCO_3_, the *Dr*-Nb(III) and apo-*Dr*-Nb solutions were allowed to equilibrate for at least 5 min; then, the pH was readjusted to the desired pH if needed [25,27,28,30,36,39].

Peroxynitrite isomerization by *Dr*-Nb(III) and apo-*Dr*-Nb was investigated via rapid mixing of the *Dr*-Nb(III) and apo-*Dr*-Nb solutions (final concentration ranging between 5.0 × 10^−6^ M and 3.5 × 10^−5^ M) with the peroxynitrite solution (final concentration ranging between 2.5 × 10^−5^ M and 2.0 × 10^−4^ M); no gaseous phase was present. Peroxynitrite isomerization was monitored at 302 nm [9,12,25,26,27,30,36,39] by the SFM-20/MOS-200 rapid-mixing stopped-flow apparatus (BioLogic Science Instruments, Claix, France); the light path of the observation chamber was 10 mm and the dead-time was 1.3 ms.

Values of the pseudo-first-order rate constant for peroxynitrite isomerization by *Dr*-Nb(III) and apo-*Dr*-Nb (i.e., *k*_obs_) were determined according to Equation (1) [13,22,23,24,25,26,27,28,29,30,31,32,33,34,35,36,37,38,39,40,41,54,55]:

[peroxynitrite]*_t_* = [peroxynitrite]*_i_* × e ^− *k*obs × *t*^ (1)

Values of the second-order rate constant for peroxynitrite isomerization by *Dr*-Nb(III) (i.e., *k*_on_), and of the first-order rate constant for the spontaneous decay of peroxynitrite (i.e., *k*_0_), were obtained from the dependence of *k*_obs_ on the *Dr*-Nb(III) concentration, according to Equation (2) [13,22,23,24,25,26,27,28,29,30,31,32,33,34,35,36,37,38,39,40,41,54,55]:

*k*_obs_ = *k*_on_ × [*Dr*-Nb(III)] + *k*_0_ (2)

The reaction of peroxynitrite with L-tyrosine was investigated at pH 7.0 and 22.0 °C, as reported elsewhere. The relative nitro-L-tyrosine yield (%) corresponds to: (yield with added *Dr*-Nb(III) or apo-*Dr*)/(yield with no *Dr*-Nb(III) or apo-*Dr*-Nb) × 100 [13,25,30,34,35,36,39,55,56].

The percentage of NO_3_^−^ and NO_2_^−^ obtained from peroxynitrite isomerization was determined spectrophotometrically at 543 nm by using the Griess reagent and vanadium(III) chloride (VCl_3_) to catalyze the conversion of NO_3_^−^ to NO_2_^−^, according to literature [25,30,36,39,56].

Kinetic data were analyzed using the GraphPad Prism program, version 5.03 (GraphPad Software, San Diego, CA, USA). The results are given as the mean values of at least four experiments plus and minus the standard deviation. Data were analyzed using GraphPad Prism 6 (GraphPad Software Inc., San Diego, CA, USA) and were expressed as mean values ± standard deviation (SD). Statistical analysis was performed using either the Student’s *t*-test or the one-way ANOVA comparison test (* *p* < 0.05; ** *p* < 0.01; *** *p* < 0.001; **** *p* < 0.0001).

## 3. Results and Discussion

In the absence and presence of CO_2_, the absorbance at 302 nm decreases upon mixing the *Dr*-Nb(III), apo-*Dr*-Nb, and peroxynitrite solutions over the whole pH range explored. According to the literature [13,22,23,24,25,26,27,28,29,30,31,32,33,34,35,36,37,38,39,40,41,54,55], this reflects peroxynitrite isomerization. No absorbance spectroscopic changes were observed in the Soret region.

**Figure 1:**
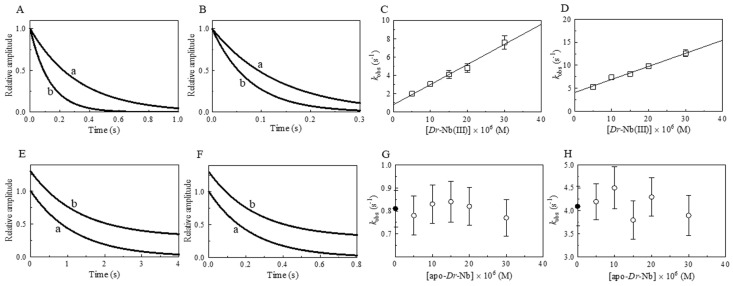
Effect of *Dr*-Nb(III) on the kinetics for peroxynitrite isomerization, at pH 5.8 and 22.0 °C. (**A**) Averaged time courses of the *Dr*-Nb(III)-catalyzed isomerization of peroxynitrite in the absence of CO_2_. The time course analysis, according to Equation (1), allowed us to determine the following values of *k*_obs_: trace a, 3.1 s^−1^, *R* = 0.998; trace b, 7.6 s^−1^, *R* = 0.998. The *Dr*-Nb(III) concentration was: trace a, 1.0×10^−5^ M; trace b, 3.0 × 10^−5^ M. (**B**) Averaged time courses of the *Dr*-Nb(III)-catalyzed isomerization of peroxynitrite in the presence of CO_2_. The time course analysis, according to Equation (1), allowed us to determine the following values of *k*_obs_: trace a, 7.4 s^−1^, *R* = 0.999; trace b, 1.3 × 10^1^ s^−1^, *R* = 0.998. The *Dr*-Nb(III) concentration was: trace a, 1.0 × 10^−5^ M; trace b, 3.0 × 10^−5^ M. The CO_2_ concentration was 1.2 × 10^−3^ M. (**C**) Dependence of *k*_obs_ on the concentration of *Dr*-Nb(III) in the absence of CO_2_. Data were analyzed according to Equation (2) with *k*_on_ = 2.2 × 10^5^ M^−1^ s^−1^ and *k*_0_ = 8.1 × 10^−1^ s^−1^; *R* = 0.989, *p* = 0.0006. (**D**) Dependence of *k*_obs_ on the concentration of *Dr*-Nb(III) in the presence of CO_2_. Data obtained were analyzed according to Equation (2) with *k*_on_ = 2.8 × 10^5^ M^−1^ s^−1^ and *k*_0_ = 4.1 s^−1^; *R* = 0.991, *p* = 0.0005. (**E**) Averaged time courses of peroxynitrite isomerization by apo-*Dr*-Nb in the absence of CO_2_. The time course analysis, according to Equation (1), allowed us to determine the following values of *k*_obs_: trace a, 8.3 × 10^−1^ s^−1^; trace b, 7.7 × 10^−1^ s^−1^. The apo-*Dr*-Nb concentration was: trace a, 1.0 × 10^−5^ M; trace b, 3.0 × 10^−5^ M. For clarity, trace b has been up-shifted of 0.3 units. (**F**) Averaged time courses of peroxynitrite isomerization by apo-*Dr*-Nb in the presence of CO_2_. The time course analysis, according to Equation (1), allowed us to determine the following values of *k*_obs_: trace a, 4.3 s^−1^; trace b, 3.9 s^−1^. The apo-*Dr*-Nb concentration was: trace a, 1.0 × 10^−5^ M; trace b, 3.0 × 10^−5^ M. For clarity, trace b has been up-shifted of 0.3 units. The CO_2_ concentration was 1.2 × 10^−3^ M. (**G**) Dependence of *k*_obs_ on the concentration of apo-*Dr*-Nb in the absence of CO_2_. The symbol on the ordinate indicates the value of *k*_0_: 8.0 × 10^−1^ s^−1^. The average value of *k*_obs_ obtained in the presence of apo-*Dr*-Nb is: 8.1 × 10^−1^ s^−1^. (**H**) Dependence of *k*_obs_ on the concentration of apo-*Dr*-Nb in the presence of CO_2_. The symbol on the ordinate indicates the value of *k*_0_: 4.1 s^−1^. The average value of *k*_obs_ obtained in the presence of apo-*Dr*-Nb and CO_2_ is: 4.1 s^−1^. In panels G and H, the differences among the *k*_obs_ values are not statistically significant. The peroxynitrite concentration was 2.0 × 10^−4^ M. The HCO_3_^−^ concentration was 5.0 × 10^−1^ M. Where not shown, the standard deviation is smaller than the symbol.

**Figure 2:**
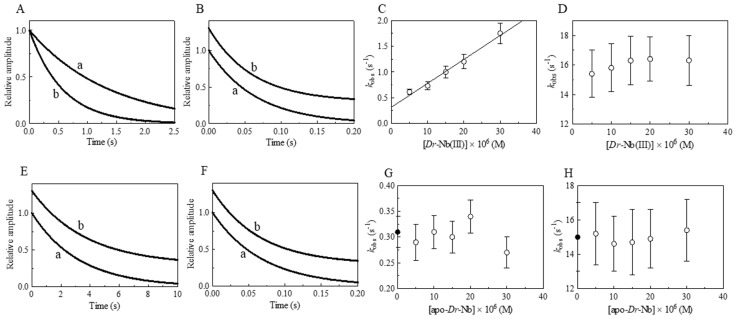
Effect of *Dr*-Nb(III) on the kinetics for peroxynitrite isomerization, at pH 7.0 and 22.0 °C. (**A**) Averaged time courses of the *Dr*-Nb(III)-catalyzed isomerization of peroxynitrite in the absence of CO_2_. The time course analysis according to Equation (1) allowed us to determine the following values of *k*_obs_: trace a, 7.3 × 10^−1^ s^−1^, *R* = 0.998; trace b, 1.8 s^−1^, *R* = 0.999. The *Dr*-Nb(III) concentration was: trace a, 1.0 × 10^−5^ M; trace b, 3.0 × 10^−5^ M. (**B**) Averaged time courses of the *Dr*-Nb(III)-catalyzed isomerization of peroxynitrite in the presence of CO_2_. The time course analysis according to Equation (1) allowed us to determine the following values of *k*_obs_: trace a, 1.5 × 10^1^ s^−1^, *R* = 0.999; trace b, 1.7 × 10^1^ s^−1^, *R* = 0.998. The *Dr*-Nb(III) concentration was: trace a, 1.0 × 10^−5^ M; trace b, 3.0 × 10^−5^ M. The CO_2_ concentration was 1.2 × 10^−3^ M. For clarity, trace b has been up-shifted of 0.3 units. (**C**) Dependence of *k*_obs_ on the concentration of *Dr*-Nb(III) in the absence of CO_2_. Data were analyzed according to Equation (2) with *k*_on_ = 4.7 × 10^4^ M^−1^ s^−1^ and k_0_ = 3.1 × 10^−1^ s^−1^; *R* = 0.989, *p* = 0.0006. (**D**) Dependence of *k*_obs_ on the concentration of *Dr*-Nb(III) in the presence of CO_2_. (**E**) Averaged time courses of peroxynitrite isomerization by apo-*Dr*-Nb in the absence of CO_2_. The time course analysis according to Equation (1) allowed us to determine the following values of *k*_obs_: trace a, 3.1 × 10^−1^ s^−1^; trace b, 2.7 × 10^−1^ s^−1^. The apo-*Dr*-Nb concentration was: trace a, 1.0 × 10^−5^ M; trace b, 3.0 × 10^−5^ M. For clarity, trace b has been up-shifted of 0.3 units. (**F**) Averaged time courses of peroxynitrite isomerization by apo-*Dr*-Nb in the presence of CO_2_. The time course analysis according to Equation (1) allowed us to determine the following values of *k*_obs_: trace a, 1.5 × 10^1^ s^−1^; trace b, 1.6 × 10^1^ s^−1^. The apo-*Dr*-Nb concentration was: trace a, 1.0 × 10^−5^ M; trace b, 3.0 × 10^−5^ M. For clarity, trace b has been up-shifted of 0.3 units. The CO_2_ concentration was 1.2 × 10^−3^ M. (**G**) Dependence of *k*_obs_ on the concentration of apo-*Dr*-Nb in the absence of CO_2_. The symbol on the ordinate indicates the value of *k*_0_: 3.1 × 10^−1^ s^−1^. The average value of *k*_obs_ obtained in the presence of apo-*Dr*-Nb is: 3.0 × 10^−1^ s^−1^. (**H**) Dependence of *k*_obs_ on the concentration of apo-*Dr*-Nb in the presence of CO_2_. The symbol on the ordinate indicates the value of *k*_0_: 1.5 × 10^1^ s^−1^. The average value of *k*_obs_ obtained in the presence of apo-*Dr*-Nb and CO_2_ is: 1.5 × 10^1^ s^−1^. In panels D, G, and H, the differences among the *k*_obs_ values are not statistically significant. The peroxynitrite concentration was 2.0 × 10^−4^ M. The HCO_3_^−^ concentration was 5.0 × 10^−1^ M. Where not shown, the standard deviation is smaller than the symbol.

**Figure 3:**
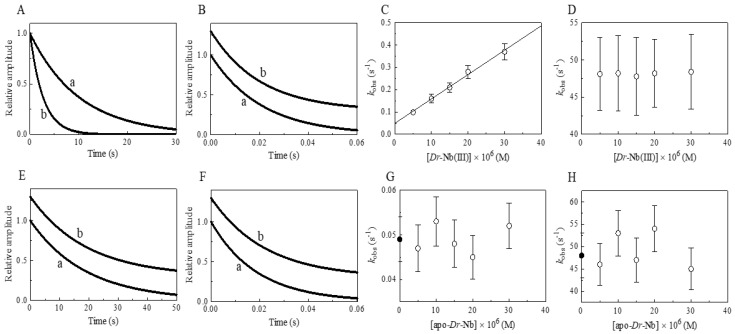
Effect of *Dr*-Nb(III) on the kinetics for peroxynitrite isomerization, at pH 8.5 and 22.0 °C. (**A**) Averaged time courses of the *Dr*-Nb(III)-catalyzed isomerization of peroxynitrite in the absence of CO_2_. The time course analysis according to Equation (1) allowed us to determine the following values of *k*_obs_: trace a, 1.0 × 10^−1^ s^−1^, *R* = 0.998; trace b, 3.7 × 10^−1^ s^−1^, *R* = 0.999. The *Dr*-Nb(III) concentration was: trace a, 1.0 × 10^−5^ M; trace b, 3.0 × 10^−5^ M. (**B**) Averaged time courses of the *Dr*-Nb(III)-catalyzed isomerization of peroxynitrite in the presence of CO_2_. The time course analysis according to Equation (1) allowed us to determine the following values of *k*_obs_: trace a, 4.8 × 10^1^ s^−1^, *R* = 0.999; trace b, 4.8 × 10^1^ s^−1^, *R* = 0.998. The *Dr*-Nb(III) concentration was: trace a, 1.0 × 10^−5^ M; trace b, 3.0 × 10^−5^ M. The CO_2_ concentration was 1.2 × 10^−3^ M. For clarity, trace b has been up-shifted of 0.3 units. (**C**) Dependence of *k*_obs_ on the concentration of *Dr*-Nb(III) in the absence of CO_2_. Data were analyzed according to Equation (2) with values of *k*_on_ = 1.1 × 10^4^ M^−1^ s^−1^ and *k*_0_ = 4.9 × 10^−2^; *R* = 0.995, *p* = 0.0002. (**D**) Dependence of *k*_obs_ on the concentration of *Dr*-Nb(III) in the presence of CO_2_. (**E**) Averaged time courses of peroxynitrite isomerization by apo-*Dr*-Nb in the absence of CO_2_. The time course analysis according to Equation (1) allowed us to determine the following values of *k*_obs_: trace a, 5.3 × 10^−2^ s^−1^; trace b, 5.2 × 10^−2^ s^−1^. The apo-*Dr*-Nb concentration was: trace a, 1.0 × 10^−5^ M; trace b, 3.0 × 10^−5^ M. For clarity, trace b has been up-shifted of 0.3 units. (**F**) Averaged time courses of peroxynitrite isomerization by apo-*Dr*-Nb in the presence of CO_2_. The time course analysis according to Equation (1) allowed us to determine the following values of *k*_obs_: trace a, 5.3 × 10^1^ s^−1^; trace b, 4.5 × 10^1^ s^−1^. The apo-*Dr*-Nb concentration was: trace a, 1.0 × 10^−5^ M; trace b, 3.0 × 10^−5^ M. For clarity, trace b has been up-shifted of 0.3 units. The CO_2_ concentration was 1.2 × 10^−3^ M. (**G**) Dependence of *k*_obs_ on the concentration of apo-*Dr*-Nb in the absence of CO_2_. The symbol on the ordinate indicates the value of *k*_0_: 4.9 × 10^−2^ s^−1^. The average value of *k*_obs_ obtained in the presence of apo-*Dr*-Nb is: 4.9 × 10^−2^ s^−1^. (**H**) Dependence of *k*_obs_ on the concentration of apo-*Dr*-Nb in the presence of CO_2_. The symbol on the ordinate indicates the value of *k*_0_: 4.8 × 10^1^ s^−1^. The average value of *k*_obs_ obtained in the presence of apo-*Dr*-Nb and CO_2_ is 4.9 × 10^1^ s^−1^. In panels D, G, and H the differences among the *k*_obs_ values are not statistically significant. The peroxynitrite concentration was 2.0 × 10^−4^ M. The HCO_3_^−^ concentration was 5.0 × 10^−1^ M. Where not shown, the standard deviation is smaller than the symbol.

Under all the experimental conditions, the time course of peroxynitrite isomerization in the absence and presence of *Dr*-Nb(III), apo-*Dr*-Nb, and CO_2_ was fitted to a single-exponential decay for more than 93% of its course, according to Equation (1) (Figure 1, Figure 2 and Figure 3, panels A and B). The values of the pseudo-first-order rate constant for peroxynitrite isomerization catalyzed by *Dr*-Nb(III) (i.e., *k*_obs_) increase with the heme protein concentration (Figure 1, Figure 2 and Figure 3, panels C and D). Previous experiments have shown that, once bound to a heme protein (likely as Fe(III)-ONOOH), peroxynitrite isomerizes to NO_3_^−^ at a rate of ~70 s^−1^ [13]. Therefore, since the values of *k*_obs_ range between 1 × 10^−1^ s^−1^ and 8 s^−1^ (Figure 1, Figure 2 and Figure 3, panel C), they indicate that, under our conditions, the formation of the *Dr*-Nb(III)-OONOH species represents the rate-limiting step of the catalytic process. Notably, the *Dr*-Nb(III)-OONOH conversion to *Dr*-Nb(III) and NO_3_^−^ is faster by at least 10-fold than the formation of the *Dr*-Nb(III)-OONOH complex.

The analysis of the data shown in Figure 1, Figure 2 and Figure 3 (panels C and D), according to Equation (2), allowed us to determine the values of *k*_on_ and *k*_0_, corresponding to the slope and the *y*-intercept of the linear plots, respectively (Table 1). Moreover, the values of *k*_0_ were measured in the absence of *Dr*-Nb(III) via the rapid mixing of the peroxynitrite solution with the appropriate Bis-Tris propane buffer solution (Figure 1, Figure 2 and Figure 3, panels E and F). Both in the absence and presence of CO_2_, the values of *k*_0_ obtained by the different methods match well with each other (Table 1) and agree well with those previously reported [12,13,25,27,28,30,33,34,36,39,54] (see Table 2).

As shown in Table 1, the values of *k*_on_ for the interaction between *Dr*-Nb(III) and peroxynitrite were unaffected by CO_2_/HCO_3_^−^/CO_3_^2−^, suggesting that CO_2_/HCO_3_^−^/CO_3_^2−^ does not alter the binding properties of *Dr*-Nb(III). The values of *k*_on_ for peroxynitrite isomerization via all-α-helical globins and all-β-barrel nitrobindins ranged between 1.2 × 10^4^ M^−1^ s^−1^ for *Hs*-Hb(III) [25] and 6.9 × 10^4^ M^−1^ s^−1^ for *Mt*-Nb(III) [40] (Table 2), suggesting that the very different structural organization [40,57,58,59,60,61] is not at the root of the different rate of peroxynitrite isomerization. However, the coordination of the heme-Fe(III) atom, the in- or out-of-plane position of the metal with respect to the pyrrole nitrogen atoms of the porphyrin, the ligand accessibility of the heme-Fe(III) atom, and its Lewis acidity may tune the kinetics of the related peroxynitrite decomposition [36].

**Figure 4:**
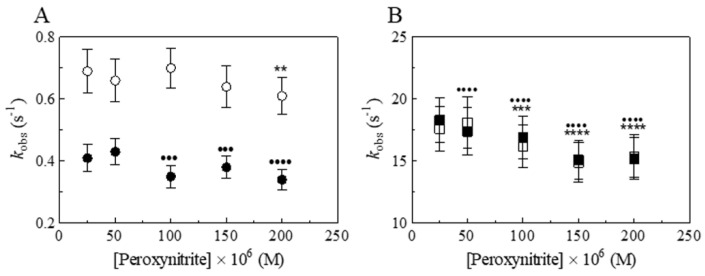
Effect of peroxynitrite concentration on the values of *k*_obs_ for the isomerization of peroxynitrite by *Dr*-Nb(III) and apo-*Dr*-Nb in the absence and presence of CO_2_, at pH 7.0 and 22.0 °C. (**A**) Dependence of *k*_obs_ on the peroxynitrite concentration in the presence of *Dr*-Nb(III) (open circles) and apo-*Dr*-Nb (filled circles), in the absence of CO_2_ (One-way ANOVA: open circles, ** *p* < 0.01, 25 × 10^−6^ M versus 200 × 10^−6^ M; filled circles, ^●●●^ *p* < 0.001, 25 × 10^−6^ M versus 100 × 10^−6^ M and versus 150 × 10^−6^ M, ^●●●●^ *p* < 0.0001, 25 × 10^−6^ M versus 200 × 10^−6^ M). (**B**) Dependence of *k*_obs_ on the peroxynitrite concentration in the presence of *Dr*-Nb(III) (open squares) and apo-*Dr*-Nb (filled squares), in the presence of CO_2_ (One-way ANOVA: open squares, *** *p* < 0.001, 25 × 10^−6^ M versus 100 × 10^−6^ M, **** *p* < 0.0001, 25 × 10^−6^ M versus 150 × 10^−6^ M and versus 200 × 10^−6^ M; filled squares, ^●●●●^ *p* < 0.0001, 25 × 10^−6^ M versus 50 × 10^−6^ M, 100 × 10^−6^ M, 150 × 10^−6^ M, and 200 × 10^−6^ M). The *Dr*-Nb(III) and apo-*Dr*-Nb(III) concentration was 5.0 × 10^−6^ M. The HCO_3_^−^ concentration was 5.0 × 10^−1^ M.

To outline the role of the metal center, the values of *k*_obs_ have been determined as a function of (i) apo-*Dr*-Nb concentration (at a fixed peroxynitrite concentration) and (ii) peroxynitrite concentration (at fixed *Dr*-Nb(III) concentration). Apo-*Dr*-Nb does not induce the isomerization of peroxynitrite; in fact, the values of *k*_obs_ and *k*_0_ match each other in the presence of apo-*Dr*-Nb (Figure 1, Figure 2 and Figure 3, panels E–H), as reported for apo-*Efc*-Mb, apo-*Hs*-Hb, and apo-*Hs*-Nb [25,39]. As shown in Figure 4, the values of *k*_obs_ slightly decrease with an increasing peroxynitrite concentration, reflecting either the slow isomerization process of peroxynitrite-peroxynitrous acid dimers or their slow dissociation preceding the *Dr*-Nb(III)-catalyzed isomerization of peroxynitrite [30,36].

As shown in Figure 1, Figure 2 and Figure 3 (panel D) and in Figure 4 (panel B) it turns out that, in the presence of 5.0 × 10^−1^ M CO_2_/HCO_3_^−^/CO_3_^2−^, the role of *Dr*-Nb(III) in characterizing the rate constant of peroxynitrite isomerization becomes progressively less relevant as the pH is raised since values of *k*_0_ for peroxynitrite isomerization obtained in the presence of CO_2_/HCO_3_^−^/CO_3_^2−^ are faster by about two orders of magnitude than those obtained in its absence (Table 1). This indicates that CO_2_/HCO_3_^−^/CO_3_^2−^ dramatically speeds up the decay of peroxynitrite through direct interaction, without interfering with peroxynitrite binding to *Dr*-Nb(III) [9,12,13,25,27,28,30,32,33,34,36].

To clarify the differential roles of various ferric heme proteins and CO_2_/HCO_3_^−^/CO_3_^2−^ levels in peroxynitrite detoxification, the overall effect of pH and CO_2_/HCO_3_^−^/CO_3_^2−^ on the observed rate of peroxynitrite isomerization was investigated. Thus, the protonation equilibria of OONO^−^/HOONO and CO_2_/HCO_3_^−^/CO_3_^2−^ were considered. The effect of CO_2_ on peroxynitrite isomerization in the absence of heme proteins (i.e., *k*_obs_) can be described by Equation (3):

(3) $$\begin{array}{c} k_{\mathrm{obs}}=\left(\frac{K_{P}}{\left[H^{+}\right]+K_{P}}\right)\times \left\{k_{0}+\left[L\right]\times \left(\frac{k_{c1}\times {\left[H^{+}\right]}^{2}+k_{c2}\times K_{c1}\times \left[H^{+}\right]+k_{c3}\times K_{c1}\times K_{c2}}{{\left[H^{+}\right]}^{2}+K_{c1}\times \left[H^{+}\right]+K_{c1}\times K_{c1}}\right)\right\}+\\ +\left(\frac{\left[H^{+}\right]}{\left[H^{+}\right]+K_{p}}\right)\times \left\{k_{0}^{H}+\left[L\right]\times \left(\frac{k_{c1}^{H}\times {\left[H^{+}\right]}^{2}+k_{c2}^{H}\times K_{c1}\times \left[H^{+}\right]+k_{c3}^{H}\times K_{c1}\times K_{c2}}{{\left[H^{+}\right]}^{2}+K_{c1}\times \left[H^{+}\right]+K_{c1}\times K_{c2}}\right)\right\} \end{array}$$

where *k*_0_ and *k*_0_^H^ are the intrinsic degradation rates of OONO^−^ and HOONO, respectively, [H^+^] (= 10^−pH^) is the proton concentration, *K*_P_ (= ([OONO^−^] × [H^+^])/[HOONO]) is the protonation constant of peroxynitrite, [L] is the reactant concentration (in our case [HCO_3_^−^] = 5.0 × 10^−^^1^ M), *K*_c1_ ([H_2_O]) (= ([HCO_3_^−^] × [H^+^])/[CO_2_] = 10^−6.34^) is the protonation equilibrium constant between HCO_3_^−^ and CO_2_, *K*_c2_ (= ([CO_3_^2−^] × [H^+^])/[HCO_3_^−^] = 10^−10.25^) is the protonation equilibrium constant between CO_3_^2−^ and HCO_3_^−^; *k*_c1_ and *k*_c1_^H^ are the second-order rate constants for the reaction of CO_2_ with OONO^−^ and HOONO, respectively, *k*_c2_ and *k*_c2_^H^ are the second-order rate constants for the reaction with HCO_3_^−^ of OONO^−^ and HOONO, respectively, and *k*_c3_ and *k*_c3_^H^ are the second-order rate constants for the reaction with CO_3_^2−^ of OONO^−^ and HOONO, respectively.

**Figure 5:**
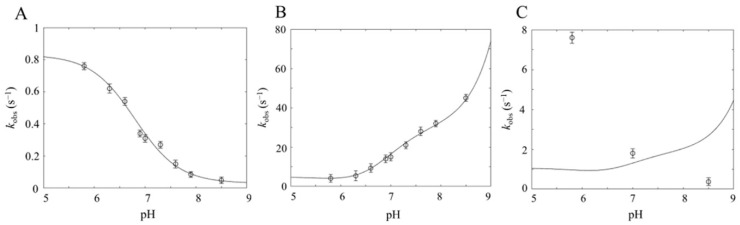
pH dependence of the peroxynitrite isomerization rate constant in the absence of CO_2_/HCO_3_^−^/CO_3_^2−^ (**A**), in the presence of 5.0 × 10^−1^ M CO_2_/HCO_3_^−^/CO_3_^2−^ (**B**), and in the presence of 3.0 × 10^−2^ M CO_2_/HCO_3_^−^/CO_3_^2−^ (**C**). In all three panels, the continuous lines correspond to *k*_obs_, as defined in Equation (3), under different conditions (i.e., [L] = 0 (**A**), [L] = 5.0 × 10^−1^ M (**B**), [L] = 3.0×10^−2^ M (**C**), employing the parameters reported in Table 3. In (**A**,**B**), the open circles correspond to experimental data obtained from the literature [36] on the peroxynitrite isomerization rate constants as a function of pH under the conditions described above. The continuous lines were obtained according to Equation (3) by the non-linear least-squares fitting of data. In (**C**), the open circles correspond to values of *k*_obs_ in the presence of 3.0 × 10^−5^ M *Dr*-Nb(III) in the absence of CO_2_/HCO_3_^−^/CO_3_^2−^ at different pH values (present study). Data shown in (**C**) indicate that, at pH 5.8, the efficiency of the heme protein (at the indicated concentration) is higher than that of the CO_2_/HCO_3_^−^/CO_3_^2−^ system, while at pH 8.5, the efficiency is lower.

The pH-dependence of peroxynitrite degradation in the absence of CO_2_/HCO_3_^−^/CO_3_^2−^ (i.e., [L] = 0, see Equation (3)), allowed us to determine the values of *k*_0_ (= 3.0 × 10^−2^ s^−1^), *k*_0_^H^ (= 8.3 × 10^−1^ s^−1^) and *K*_P_ (= 10^−6.8^) (Figure 5, panel A), showing that HOONO decays to NO_3_^−^ faster than OONO^−^, with a p*K*_a_ ≈ 6.8 [13,36]. On the other hand, as already outlined before [9,13,22,27,28,30,36,39], when CO_2_/HCO_3_^−^/CO_3_^2−^ is present (i.e., [L] = 5.0 × 10^−^^1^ M), the rate of peroxynitrite degradation becomes much faster and increases with a rising pH (see Table 1 and [36]). Therefore, employing Equation (3) and knowing the values of *K*_c1_ and *K*_c2_ (see above), the pH dependence of peroxynitrite degradation (Figure 5, panel B) gives information on the different values of *k*_ci_ and *k*_ci_^H^ (with i = 1, 2, 3). The inspection of Table 3 allows for the following considerations:

(i) *k*_c1_, *k*_c2_^H^, and *k*_c3_^H^ are not playing any role, likely because either one or both reactants are too scarcely populated over the pH range investigated for the pseudo-first-order rate constant to have a detectable value, which can then be considered as ≈0 s^−1^;

(ii) At pH ≤ 6.2, the peroxynitrite degradation rate is mostly characterized by the reaction between HOONO and CO_2_, corresponding to *k*_c1_^H^ = 8.0 M^−1^ s^−1^);

(iii) For 6.2 < pH < 8.2, the peroxynitrite degradation rate is mostly characterized by the reaction between OONO^−^ and HCO_3_^−^, corresponding to *k*_c2_ = 6.2 × 10^1^ M^−1^ s^−1^;

(iv) For pH ≥ 8.2, the peroxynitrite degradation rate is mostly characterized by the reaction between OONO^−^ and CO_3_^2−^, corresponding to *k*_c3_ = 1.8 × 10^3^ M^−1^ s^−1^.

**Scheme 2:**
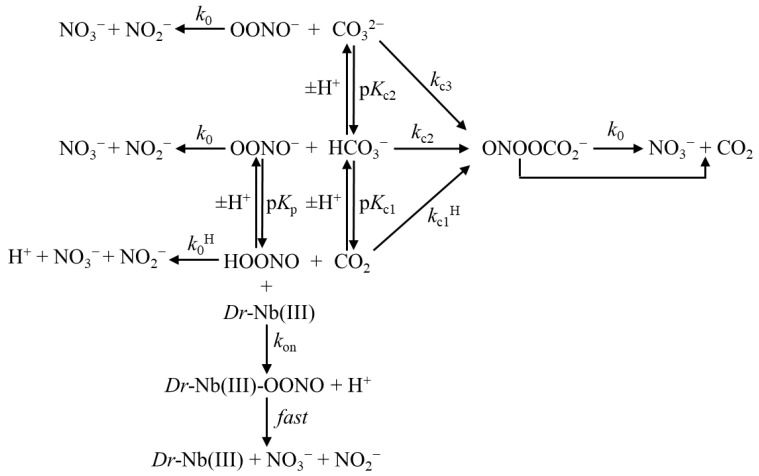
Effect of pH on peroxynitrite degradation. The scheme reproduces the mechanism described by Equation (3), clarifying the meaning of the different parameters; only the significant parameters are reported (see text).

Obviously, the value of *k*_obs_ and its pH dependence depend on the CO_2_/HCO_3_^−^/CO_3_^2−^ levels (i.e., [L]), envisaging that the relative levels of the ferric heme protein and CO_2_/HCO_3_^−^/CO_3_^2−^ are crucial in defining the respective role for peroxynitrite detoxification. Of note, data reported in Figure 1, Figure 2 and Figure 3 (panel D) refer to [CO_2_/HCO_3_^−^/CO_3_^2−^] = 5.0 × 10^−1^ M, while in the bloodstream, the physiological levels of CO_2_/HCO_3_^−^/CO_3_^2−^ (~3.0 × 10^−2^ M) are about 10-20 fold lower. Therefore, under these conditions, *k*_obs_ decreases significantly even in the presence of CO_2_/HCO_3_^−^/CO_3_^2−^, as indicated by Equation (3) (Figure 5, panel C). The pH-dependent mechanism of peroxynitrite degradation can be described by Scheme 2, which reports the different pathways for peroxynitrite degradation.

According to Equation (3), the effects of the heme protein and CO_2_/HCO_3_^−^/CO_3_^2−^ levels were simulated (Figure 5, panel C), with the values of the rates at [CO_2_/HCO_3_^−^/CO_3_^2−^] = 3.0 × 10^−2^ M and [*Dr*-Nb(III)] = 3.0 × 10^−5^ M (see Figure 1, Figure 2 and Figure 3, panels C). At acidic pH values, the role of *Dr*-Nb(III) (= 3.0 × 10^−5^ M) is prevalent for peroxynitrite isomerization, while at a neutral pH the role of *Dr*-Nb(III) (= 3.0 × 10^−5^ M) is equivalent to that of the CO_2_/HCO_3_^−^/CO_3_^2−^ system (Figure 5C). For alkaline pH values, *Dr*-Nb(III) levels higher than 3.0 × 10^−5^ M would be required to play a relevant role in peroxynitrite detoxification (see Figure 5, panel C).

**Figure 6:**
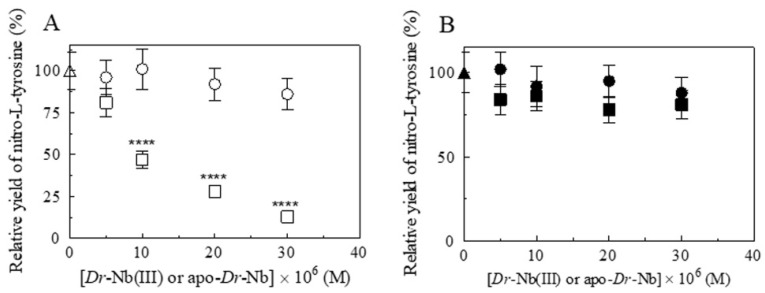
Effect of *Dr*-Nb on L-tyrosine nitrosylation induced from peroxynitrite, at pH 7.0 and 22.0 °C. (**A**) Dependence of the nitro-L-tyrosine yield on the *Dr*-Nb(III) (open squares) and apo-*Dr*-Nb (open circles) concentration in the absence of CO_2_ (One-way ANOVA: open squares, **** *p* < 0.0001, 0 M versus 10 × 10^−6^ M, 20 × 10^−6^ M, and 30 × 10^−6^ M). (**B**) Dependence of the nitro-L-tyrosine yield on the *Dr*-Nb(III) (filled squares) and apo-*Dr*-Nb (filled circles) concentration in the presence of CO_2_. The symbols on the *y*-axis (open and filled triangle) indicate the nitro-L-tyrosine yield in the absence and presence of CO_2_, respectively. The L-tyrosine concentration was 1.0 × 10^−4^ M. The peroxynitrite concentration was 2.0 × 10^−4^ M. Where not shown, the standard deviation is smaller than the symbol.

The relative importance of the heme proteins and CO_2_/HCO_3_^−^/CO_3_^2−^ catalyzing the peroxynitrite isomerization is crucial since, according to the literature [9,12,25,30,36,62,63], ferric heme proteins prevent L-tyrosine nitration, which, instead, occurs either in the presence of apo-*Dr*-Nb and/or of CO_2_/HCO_3_^−^/CO_3_^2−^ (Figure 6) (see also Scheme 1). In fact, the relative yield of NO_3_^−^ and NO_2_^−^, obtained from peroxynitrite isomerization catalyzed by *Dr*-Nb(III), ranged between 89 and 92%, and between 7 and 12%, respectively. However, in the absence of *Dr*-Nb(III) and/or in the presence of apo-*Dr*-Nb and/or CO_2_/HCO_3_^−^/CO_3_^2−^, the values of the relative yield of NO_3_^−^ and NO_2_^−^ ranged between 69 and 74%, and between 7 and 12%, respectively (Table 4). The great relevance of these observations is confirmed by the fact that, under stressed conditions, zebrafish triggers a defense mechanism through nitrosative stress and peroxynitrite production for which its degradation may be, on one side, enhanced by the elevated levels of CO_2_ [48,49], but, on the other side, inhibited by the consequent lowering of the pH level. In any event, the correlation between the peroxynitrite degradation by ferric heme proteins and CO_2_ levels appears to be relevant within in vivo models of zebrafish, and it may have important consequences for the O_2_ supply to poorly oxygenated tissues, such as the retina [50,51].

## 4. Conclusions

Ferric heme proteins and the CO_2_/HCO_3_^−^/CO_3_^2−^ system are the major players in peroxynitrite detoxification, for which efficiency depends on both the concentration of these two actors and the pH level. CO_3_^2−^ is much more effective at peroxynitrite inactivation, as compared to CO_2_, with HCO_3_^−^ displaying an intermediate activity. Of note, the CO_3_^2−^ levels were much lower than those of the other two components in the system under physiological conditions. Although ferric heme proteins are intrinsically much more effective than the CO_2_/HCO_3_^−^/CO_3_^2−^ system, their levels are usually significantly lower than CO_2_/HCO_3_^−^/CO_3_^2−^. Since over the physiological pH range the rate of peroxynitrite detoxification by CO_2_/HCO_3_^−^/CO_3_^2−^ (i.e., ~3.0 × 10^−2^ M) is ~1.5 s^−1^, values of *k*_obs_ (= *k*_on_ × [heme-Fe(III)]), for peroxynitrite scavenging by ferric heme-proteins must be larger than 2 s^−1^. Overall, only the level of the circulating *Hs*-Hb(III) (~2.0 × 10^−4^ M; corresponding to about 2–3% of the total *Hs*-Hb in red cells) appears to be sufficient to play a relevant role in the detoxification by peroxynitrite under physiological conditions.

Furthermore, it must be remarked that acidification, which often occurs in the bloodstream of fish when they dive to depth [64,65,66] and can even be magnified by increased levels of CO_2_ [49], decreases the effect of the CO_2_/HCO_3_^−^/CO_3_^2−^ system, rendering the role of ferric heme proteins even more crucial. This is especially important in the ocular system and in the protection of the retina against oxidative stress linked to peroxynitrite [3,50]. Therefore, under specific environmental conditions (typical of diving fish), *Dr*-Nb(III) indeed may play a relevant physiological role in peroxynitrite scavenging from poorly oxygenated tissues, such as the retina.

## Figures and Tables

**Table 1:** Effect of pH and CO_2_ on the values of *k*_on_ and *k*_0_ for *Dr*-Nb(III)-induced isomerization of peroxynitrite at 22.0 °C ^a^.

**pH**	**− CO_2_**		**+ CO_2_**	
	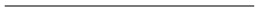		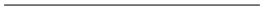	
	*k*_on_ (M^−1^ s^−1^)	*k*_0_ (s^−1^)	*k*_on_ (M^−1^ s^−1^)	*k*_0_ (s^−1^)
5.8	(2.2 ± 0.3) × 10^5^	(8.1 ± 0.8) × 10^−1^	(2.8 ± 0.3) × 10^5^	4.1 ± 0.4
7.0	(4.7 ± 0.5) × 10^4^	(3.1 ± 0.3) × 10^−1^	n.d.	(1.5 ± 0.2) × 10^1^
8.5	(1.1 ± 0.1) × 10^4^	(4.9 ± 0.5) × 10^−2^	n.d.	(4.8 ± 0.5) × 10^1^

^a^ The CO_2_ concentration was 1.2 × 10^−3^ M. 5.0 × 10^−2^ M Bis-Tris propane buffer. n.d., not determined.

**Table 2:** Effect of pH and CO_2_ on values of *k*_on_ and *k*_0_ for heme-protein-induced isomerization of peroxynitrite.

**Heme-Protein**	**− CO_2_**		**+ CO_2_**	
	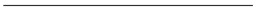		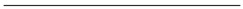	
	*k*_on_ (M^−1^ s^−1^)	*k*_0_ (s^−1^)	*k*_on_ (M^−1^ s^−1^)	*k*_0_ (s^−1^)
*Ma*-Pgb(III) ^a^	3.8 × 10^4^	2.8 × 10^−1^	n.d.	n.d.
*Mt*-trHbN(III) ^b^	6.2 × 10^4^	2.7 × 10^−1^	n.d.	n.d.
*Ph*-trHbO(III) ^c^	2.9 × 10^4^	2.8 × 10^−1^	n.d.	n.d.
*Cj*-trHbP(III) ^d^	9.6 × 10^5^	3.0 × 10^−1^	8.8 × 10^5^	2.1 × 10^1^
*Efc*-Mb(III) ^e^	2.9 × 10^4^	3.5 × 10^−1^	7.7 × 10^4^	1.7 × 10^1^
*Pc*-Mb(III) ^f^	1.6 × 10^4^	n.d.	n.d.	n.d.
*Hs*-Hb(III) ^e^	1.2 × 10^4^	3.0 × 10^−1^	3.9 × 10^4^	1.7 × 10^1^
*Mt*-Nb(III) ^g^	6.9 × 10^4^	2.6 × 10^−1^	n.d.	n.d.
*At*-Nb(III) ^g^	3.7 × 10^4^	3.0 × 10^−1^	n.d.	n.d.
*Dr*-Nb(III) ^h^	4.7 × 10^4^	3.1 × 10^−1^	n.d.	1.5 × 10^1^
*Hs*-Nb(III) ^i^	3.4 × 10^4^	2.6 × 10^−1^	n.d.	n.d.

^a^ pH 7.4 and 20.0 °C. From [55]. ^b^ pH 7.0 and 20.0 °C. From [34]. ^c^ pH 7.0 and 20.0 °C. From [35]. ^d^ pH 7.3 and 25.0 °C. From [36]. ^e^ pH 7.0 and 20.0 °C. From [25]. ^f^ pH 7.5 and 20.0 °C. From [27]. ^g^ pH 7.2 and 25.0 °C. From [40]. ^h^ pH 7.0 and 22.0 °C. Present study. ^i^ pH 7.1 and 25.0 °C. From [39]. n.d., not determined.

**Table 3:** Values of the parameters fitting data with Equation (3).

Parameter	Value
*k*_0_ (s^−1^)	(3.0 ± 0.5) × 10^−2^
*k*_0_^H^ (s^−1^)	(8.3 ± 1.0) × 10^−1^
*K*_P_ (M)	(1.6 ± 0.3) × 10^−7^
*K*_c1_ (M)	(4.6 ± 0.9) × 10^−7^
*K*_c2_ (M)	(5.6 ± 1.2) × 10^−11^
*k*_c2_ (M^−1^s^−1^)	(6.2 ± 1.3) × 10^1^
*k*_c3_ (M^−1^s^−1^)	(1.7 ± 0.4) × 10^3^
*k*_c1_^H^ (M^−1^s^−1^)	8.0 ± 2.1

**Table 4:** Percentage of NO_3_^−^ and NO_2_^−^ obtained from peroxynitrite isomerization at pH 7.0 and at 22.0 °C ^a^.

*Dr*-Nb(III)	Apo-*Dr*-Nb	CO_2_	NO_3_^−^	NO_2_^−^
(M)	(M)	(M)	(%)	(%)
0.0	0.0	0.0	73 ± 8	28 ± 3
0.0	0.0	1.2 × 10^−3^	69 ± 7	30 ± 3
0.0	3.5 × 10^−5^	0.0	71 ± 8	29 ± 3
0.0	3.5 × 10^−5^	1.2 × 10^−3^	74 ± 7	25 ± 2
3.5 × 10^−5^	0.0	0.0	89 ± 9	12 ± 2
3.5 × 10^−5^	0.0	1.2 × 10^−3^	93 ± 8	7 ± 1
3.5 × 10^−5^	3.5 × 10^−5^	0.0	91 ± 9	10 ± 1
3.5 × 10^−5^	3.5 × 10^−5^	1.2 × 10^−3^	92 ± 9	8 ± 1

^a^ The peroxynitrite concentration was 2.0 × 10^−4^ M; 5.0 × 10^−2^ M Bis-Tris propane buffer.

## Data Availability

All the data will be available upon request to the corresponding authors.
